# ﻿ *Tricosauniseriata*, a new species of xyleborine ambrosia beetle from Thailand (Coleoptera, Curculionidae, Scolytinae, Xyleborini)

**DOI:** 10.3897/zookeys.1153.101985

**Published:** 2023-03-15

**Authors:** Wisut Sittichaya, Anthony I. Cognato

**Affiliations:** 1 Agricultural Innovation and Management Division, Faculty of Natural Resources, Prince of Songkla University, Had Yai, Songkhla, 90112, Thailand Prince of Songkla University Had Yai Thailand; 2 Department of Entomology, Michigan State University, 288 Farm Lane, 243 Natural Science Bldg., East Lansing, MI 48824, USA Michigan State University East Lansing United States of America

**Keywords:** Ambrosia beetles, key, new species, Thailand, *Tricosa*, Xyleborini

## Abstract

A new species, *Tricosauniseriata***sp. nov.**, is described here. A list of *Tricosa* species found in Thailand with distributions and an updated key to *Tricosa* are also provided.

## ﻿Introduction

The xyleborine ambrosia beetle genus *Tricosa* Cognato, Smith & Beaver, 2020 (Curculionidae, Scolytinae) contains six species (Cognato et al. 2020; Smith et al. 2022). Two species were originally described as *Xyleborus* Eichhoff, 1864 and *Cyclorhipidion* Hagedorn, 1912, and four species were subsequently described (Cognato et al. 2020; Smith et al. 2022). *Tricosa* shares diagnostic characters with three xyleborine genera, *Cyclorhipidion* Hagedorn, 1912, *Cryptoxyleborus* Schedl, 1937, and *Fraudatrix* Cognato, Smith & Beaver, 2020, including either a setose and/or an attenuate appearance. *Tricosa* is distinguished from *Cyclorhipidion* by the slightly tapering elytra, from *Cryptoxyleborus* by the obliquely triangular protibial and attenuate elytra, and from *Fraudatrix* by the four-segmented antennal funicle, the type 3 antennal club with one or two sutures visible on the posterior face, and the pronotal disc being as long as or shorter than the anterior slope (Cognato et al. 2020). *Tricosa* species are mainly distributed in southern Asia and eastern Papua New Guinea (Cognato et al. 2020; Smith et al. 2020, 2022). Three species have been previously recorded from Thailand, *Tricosacattienensis* Cognato, Smith & Beaver, 2020, *T.indochinensis* Cognato, Smith & Beaver, 2020, and *T.metacuneolus* (Eggers, 1940) (Beaver et al. 2014; Cognato et al. 2020). In this present study, we describe a new species, increasing the number of Thai *Tricosa* species to four and adding a seventh member to the genus (Table 1).

**Table 1.** Synoptic list of the *Tricosa* fauna of Thailand.

Species	First record	Thai distribution
*Tricosacattienensis*	Cognato et al. 2020	N: Chiang Mai; N-E: Chaiyaphum; W: Phetchaburi; S: Surat Thani
*Tricosaindochinensis*	Cognato et al. 2020	N: Chiang Mai
*Tricosametacuneolus*	Beaver et al. 2014	S: Chumphon, Nakhon Sri Thammarat
*Tricosauniseriata* sp. nov.	This publication	S: Narathiwat

## ﻿Materials and methods

A specimen was collected from a small branch of *Artocarpusinteger* (Moraceae) in the lowland tropical rain forest of the Hala-Bala Wildlife Sanctuary, Narathiwat province, Thailand. Photographs were taken with a Canon 5D digital camera with a Canon MP-E 65 mm macro lens (Canon, Tokyo, Japan) and StackShot-Macrorail (Cognisys Inc., MI, USA) The photos were then combined with Helicon Focus 6.8.0. (Helicon Soft, Ukraine), and all photos were improved with Adobe Lightroom classic (Adobe Systems, CA, USA). The antennal and pronotum types and characters follow those proposed by Hulcr et al. (2007) and subsequently elaborated upon by Smith et al. (2020). Length was measured from the pronotal apex to the apex of the declivity, and width was measured at the widest part of the specimen.

## ﻿Results

### ﻿Taxonomic treatment

#### ﻿ Tricosa

Taxon classificationAnimaliaColeopteraCurculionidae

Cognato, Smith & Beaver, 2020

1ED6E80A-FBD3-5A44-98E8-9620BD29F6FD

##### Type species.

*Xyleborusmetacuneolus* Eggers, 1940.

##### Diagnosis.

Antennal funicle four-segmented; antennal club with one or two sutures visible on the posterior face; protibia distinctly or obliquely triangular with six or fewer denticles on outer margin and posterior face flattened and unarmed; scutellum small, flush with elytra surface; mycangial tufts absent; elytra attenuate; posterolateral costa absent.

##### Similar genera.

*Cryptoxyleborus*, *Cyclorhipidion*, and *Fraudatrix*.

#### ﻿ Tricosa uniseriata

Taxon classificationAnimaliaColeopteraCurculionidae

Sittichaya & Cognato sp. nov.

B7A495CA-64DC-5A3E-8E54-1A1EB2929648

https://zoobank.org/B97E9080-B657-4301-A775-CA9C057A644C

Fig. 1A–F

##### Type material.

***Holotype***, female, Thailand, Narathiwat Province, Hala-Bala Wildlife Sanctuary, 5°48'02.4"N, 101°49'58.2"E, lowland tropical rainforest, 140 m a.s.l., 12.x.2021, ex. small branch of *Artocarpusinteger* (W. Sittichaya) (NHMW, Naturhistorisches Museum Wien, Wien).

##### Similar species.

*T.hipparion* Smith, Beaver & Cognato, 2022.

##### Diagnosis.

This species is distinguished by its stoutness: 2.35 mm long, 2.40× as long as wide. The combination of the following characters is diagnostic: lateral margin of elytra feebly broadened apically; elytral disc convex; discal striae and interstriae uniseriate punctate; stria weakly impressed and interstriae elevated; anterior margin of pronotum with six moderate serrations.

##### Description

**(female).** 2.35 mm long, 2.40× as long as wide. Body dark brown, except appendages yellowish brown. Body more robust, less elongate. ***Head***: epistoma entire, transverse, lined with a row of short, hair-like setae. Frons flat from epistoma to middle level of eye, then slightly convex upward to vertex; lower portion of surface at medial line shagreened, subshining; sides of medial line glabrous, strongly shinning; upper portion shagreened, subshining; frons with widely separated, small granules, with each granule decorated with long, fine, hair-like setae. Eyes weakly emarginate above level of antennal insertion; upper portion of eyes slightly smaller than lower part. Submentum slightly impressed below genae, widely triangular at base. Antennal club type 3 (Hulcr et al. 2007; Smith et al. 2020); scape regularly thick, as long as club. Antennal funicle four-segmented; first segment longest and other segments approximately equal in length. Pedicel shorter than funicle. Club flattened, approximately circular (type 3) (Hulcr et al. 2007; Smith et al. 2020); segment 1 corneous, transverse on anterior face, occupying basal ~1/3; segment 2 slightly concave, corneous and corneous line very narrow; segments 1 and 2 present on posterior face.

**Figure 1.**
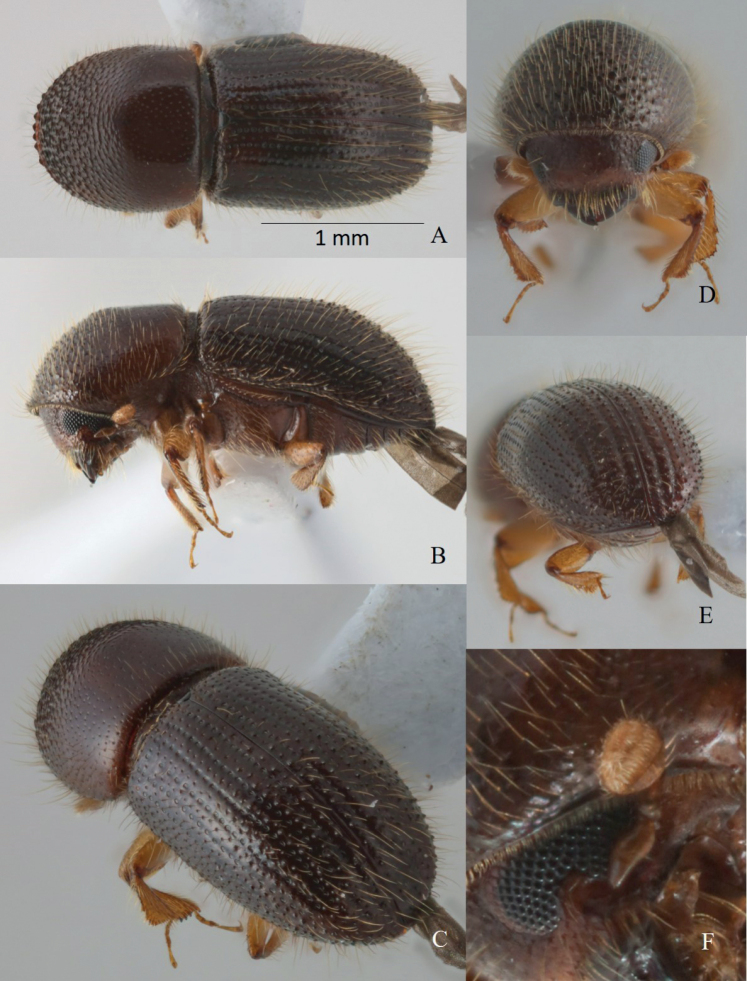
*Tricosauniseriata* sp. nov. holotype female **A** dorsal view **B** ventro-lateral view **C** posterolateral view **D** front **E** declivity **F** antenna.

***Pronotum***: 1.0× as long as wide; elongate and parallel sided in dorsal view, type 7 (Hulcr et al. 2007; Smith et al. 2020); sides parallel in basal 2/3, rounded anteriorly; anterior margin with a row of six serrations. In lateral view, elongate, disc longer than anterior slope, type 7, summit low, on apical 2/5. Surface alutaceous, anterior 1/2 asperate; asperities robust, close together, arranged in concentric arcs from midpoint of pronotum to anterior and anterolateral areas; disc evenly punctate, punctures moderate in size, round and deep, sparse and separated by glabrous, shinning areas 3–5× the size of puncture, each puncture with short, erect, hair-like seta, some longer hair-like setae at margins. Lateral margins obliquely costate. Base transverse, posterior angles acutely rounded. ***Scutellum***: small, narrow, linguiform, flush with elytra, shiny. ***Elytra***: 1.36× as long as wide, 1.44× as long as pronotum. Base transverse, margins oblique; humeral angles rounded. Sides subparallel, slightly broader from base to apical 3/4, then attenuate at apical 1/4, apex attenuate, broadly rounded. Disc convex, surface smooth, shinning, striae and interstriae uniseriate. Striae feebly impressed, punctate, punctures round, broad, and shallow, separated by 1/2 the width of a puncture, each puncture with a short, fine, inconspicuous, hair-like seta. Interstriae feebly elevated, elevation more evident apically; interstriae minutely uniseriate punctate-granulate, widely spaced, near elytral base with fine punctures and small granules on apical 1/2, granules slightly increasing in size apically; each puncture or granule with a very long, hair-like seta. Declivity occupying ~1/3 of elytra, gradual, face feebly convex, subshining; striae feebly impressed, punctate, punctures similar in size with those on the disc, each with short, fine, hair-like seta; interstriae feebly elevated, with small granules, granule apices curved ventrad, each granule with very long hair-like seta; setae on declivity twice as long as those of disc. Posterolateral margins rounded and granulate. ***Legs***: procoxae contiguous, prosternal posterocoxal piece conical, slightly inflated. Protibiae distinctly triangular, broadest at apical 1/3, posterior face flat, unarmed; outer margin armed with five large denticles at apical 1/3. Meso- and metatibiae obliquely triangular, flattened, posterior face unarmed; outer margin armed with 6 and 10 moderately socketed denticles.

**Male.** Unknown.

##### Etymology.

*L. uniseriata*: *uni*- = one; *series* = row. Refers to the arrangement of strial and interstrial punctures in one line. A variable adjective.

##### Distribution.

Thailand (Narathiwat Province).

##### Host plants.

The holotype was collected from a small branch of *Artocarpusinteger* (Thunb.) Merr. (Moraceae).

## ﻿Discussion

*Tricosauniseriata* is the smallest and stoutest *Tricosa* species. The proportion of this species’ body is 2.40× as long as wide as compared to the other species. The elytra are feebly widened 3/4 from the base and slightly tapered to apex as compared to *T.hipparion* where the elytra are parallel sided 2/3 from the base and tapered to the apex (Cognato et al. 2020; Smith et al. 2022). Its pronotum is less posteriorly elongate, which is similar to *T.hipparion* and *T.mangoensis* (Schedl, 1942), and different from the other species, which have a more posteriorly elongated pronotum. The elytral apex is less tapering, broadly rounded, and similar to *T.hipparion*.

### ﻿Key to the species of *Tricosa* (females only)

Modified from Cognato et al. (2020).

**Table:** 

1	Elytral disc slightly convex without transverse impression	**2**
–	Elytral disc deeply transversely impressed with a saddle-like depression	***T.hipparion***
2	Elytral discal striae and interstriae uniseriate punctate	**3**
–	Elytral discal striae and interstriae punctures confused	**6**
3	Pronotum anterior margin unarmed, protibia broad, appearing distinctly triangular	***T.jacula* Cognato, Smith & Beaver, 2020**
–	Pronotum anterior margin serrate, protibia narrow, appearing obliquely triangular	**4**
4	Discal striae and interstriae flat, body more elongate 2.53–2.78× as long as wide, elytra more tapering, apex broadly acute	**5**
–	Discal striae feebly impressed, interstriae feebly elevate, body shorter and stouter 2.40× as long as wide, elytra less tapering, apex broadly round	***T.uniseriata* sp. nov.**
5	Smaller in size, 2.40–2.50 mm, and declivital interstriae moderately setose	***T.metacuneolus***
–	Larger, 3.80 mm, and declivital striae and interstriae densely setose	***T.mangoensis***
6	Pronotum anterior margin armed by a row of six serrations. Smaller, 2.70–3.10 mm long, and stouter, 2.50–2.70× as long as wide	***T.cattienensis***
–	Pronotum anterior margin armed by a row of eight serrations. Larger, 3.20–3.40 mm long, and more slender, 2.83–2.91× as long as wide	***T.indochinensis***

## Supplementary Material

XML Treatment for Tricosa

XML Treatment for Tricosa uniseriata
